# *Codonopsis lanceolata* Water Extract Increases Hepatic Insulin Sensitivity in Rats with Experimentally-Induced Type 2 Diabetes

**DOI:** 10.3390/nu9111200

**Published:** 2017-11-01

**Authors:** Seong-Yeop Jeong, Suna Kang, Da Sol Kim, Sunmin Park

**Affiliations:** 1 Department of R & D, Sunchang Research Center for Fermentation Microbes, Sunchang-Gun, Sunchang-yup 56048, Korea; jsyeop78@gmail.com; 2 Department of Food & Nutrition, Obesity/Diabetes Center, Hoseo University, 165 Sechul-Ri, BaeBang-Yup Asan-Si, ChungNam-Do 3499, Korea; roypower003@naver.com (S.K.); tpfptm14@daum.net (D.S.K.)

**Keywords:** *Codonopsis lanceolata*, hepatic insulin signaling, insulin resistance, insulin secretion, type 2 diabetes

## Abstract

We examined the mechanisms and efficacy of *Codonopsis lanceolata* water extract (CLW) for treating type 2 diabetic (T2DM) symptoms. Partial pancreatectomized (Px) rats, a non-obese T2DM model, were provided high fat diets containing cellulose (control), 0.3% (CLW-L) or 1% CLW (CLW-H) for eight weeks. The positive control group was provided with rosiglitazone (20 mg/kg bw/day). The control group had lower epididymal fat masses than the CLW and the positive control groups, possibly due to urinary glucose loss, although CPT-1 and SIRT-1 expression was higher in the CLW group. CLW-H significantly reduced serum glucose levels and urinary glucose loss compared to the untreated control. The improvement of glucose utilization was associated with a higher fat mass in the CLW-H and positive control groups. Glucose-stimulated insulin secretion was higher in the untreated control than other groups and CLW tightly regulated insulin secretion as much as the positive control, and it was much tighter than the untreated control. Glucose infusion rates were higher during the hyperinsulinemic euglycemic clamp in the CLW and positive controls than the untreated control, and liver glucose outputs were lower during basal and hyperinsulinemic conditions in the CLW and positive control groups than the untreated control group. The increased hepatic insulin sensitivity was associated with enhanced insulin signaling in CLW (pAkt➔pGSK-1β). In conclusion, CLW consumption effectively alleviated diabetic symptoms by improving insulin sensitivity, potentiating hepatic insulin signaling and tightly regulating the insulin secretion capacity in non-obese T2DM rats.

## 1. Introduction

Type 2 diabetes is known to develop as a consequence on insulin secretion not compensating for insulin resistance [1]; however, the precise mechanism by which the impairment of the compensation leads to type 2 diabetes is unclear. The major pathways leading to type 2 diabetes are different in Asians and Caucasians. The International Diabetes Federation has reported that there were 113,900,000 Asian adults with type 2 diabetes in 2013, which accounted for 36% of the global total. This is expected rise to 180,000,000 by 2035 [2]. Over 60% of type 2 diabetic patients worldwide are Asians [2]. These data indicate that Asians are more susceptible to becoming diabetic, which is associated with lower β-cell function and mass when they develop insulin resistance due to aging, obesity and inflammation [3]. Asian type 2 diabetic patients tend have normal body weights which usually decline when the diabetic status is severe [3]. Thus, the goal of diabetes treatments should be to manage the balance of insulin resistance and insulin secretion, especially in Asians.

Animal models for Asian type 2 diabetes should be non-obese and have an insulin secretion capacity that is incapable of maintaining normoglycemia concurrent with insulin resistance [3]. Partial pancreatectomized (Px) rats meet these criteria for Asian type 2 diabetes [4]. They are non-obese and have about 50–60% of the insulin secretion capacity of normal rats, and they show almost normal fasting serum glucose levels and slightly higher post-prandial serum glucose levels [4]. In increased insulin resistant states, such as when fed a high fat diet, they develop the symptoms of type 2 diabetes without showing obesity. Likewise, the treatment for Asian type 2 diabetic patients should be focused on multiple targets, including insulin sensitivity, β-cell mass and insulin secretion. Combinations of insulin sensitizers and insulin secretagogues like glucagon-like peptide-1 receptor agonist have been used for treating type 2 diabetes even in Caucasians [5]. Although hypoglycemic agents acutely normalize serum glucose levels, they have some adverse effects such as hypoglycemia and β-cell failure. Herbal treatments have been suggested as alternative medicines since they have slowly improved glycemic control with minimal side effects [4].

*Codonopsis lanceolata*, a member of the *Campanulaceae* plant family, is endemic to the mountains and forests of East Asia [6]. *C. lanceolata* root has been used as a food and a traditional medicine for treating inflammation and abnormal immune-related diseases. The freeze-dried water extract of *C. lanceolata* root (CLW) does not cause acute or subchronic toxicity in rats and has a 5000 mg/kg 50% lethal dose [7]. Thus, CLW is safe, but Hur et al. [8] reported that CLW can induce severe food allergies such as anaphylaxis by increasing the release of histamine. CLW contains tannins, triterpenoid saponins (lancemaside A, lancemaside B, lancemaside E, foetidissimoside A and etc.), flavonoids, alkaloids, inulin, essential oils, and sterols, which contribute to various pharmacological activities [6]. CLW suppresses the production of proinflammatory cytokines such as TNF-α, nitric oxide, interleukin (IL)-3 and IL-6 in LPS-stimulated macrophage [9]. CLW significantly improves anti-adipogenic activity and dyslipidemia in diet-induced obese rats [10]. CLW also improves amyloid β-induced memory deficits with acetylcholinesterase inhibition, and activates cAMP-responding element binding proteins, brain-derived neurotrophic factor, and extracellular signal-regulated kinase expression in the hippocampus [11]. Since glucose dysregulation is associated with memory deficits and cognitive decline, and type 2 diabetes and Alzheimer’s disease appear to exhibit regulatory cross-talk [12], CLW may have hypoglycemic activity. Although the isolated effective components of CLW have not been studied for their efficacy for treating metabolic diseases; the saponins, flavonoids and inulin in CLW may have a hypoglycemic effect by potentiating insulin secretion and improving insulin resistance. Inulin has been reported to control serum glucose levels in type 2 diabetic patients in a systematic review and meta-analysis [13].

We hypothesized that prolonged consumption of CLW could enhance glucose homeostasis in a normal-weight insulin-insufficient animal model of type 2 diabetes. The hypothesis was tested in partial Px rats fed a high-fat diet. The anti-diabetic mechanisms of action of CLW were explored by evaluating its ability to normalize insulin sensitivity and secretion.

## 2. Methods

### 2.1. Water Extracts of C. lanceolata

*C. lanceolata* was dried, ground and then extracted in water with five times the weight at 80 °C in an ultrasonic extractor. This extract was concentrated using reflux extraction at 70 °C and supernatants were taken after centrifuging at 3000× *g* for 20 min. The supernatants were made into powder by freeze-drying. The yield of *C. lanceolata* was 19.3%.

We determined the total phenolic compounds in CLW using Folin–Ciocalteu reagent as previously described [14]. Briefly, the sample extract was mixed with an equal volume of 1 N Folin–Ciocalteu reagent and held for 3 min at 25 °C; after which a 2% solution of sodium carbonate was added and reacted for 1 h. The color changes were spectrophotometrically measured at 750 nm (Perkin-Elmer, Waltham, MA, USA). A standard curve was derived with gallic acid (Sigma Chemical Co., St. Louis, MO, USA), and the total phenolic content was expressed as mg of gallic acid equivalents (GAE) per g dry weight. Total flavonoid content in the CLW were also determined using the previously described method [15]. The CLW was mixed with a 5% solution of sodium nitrite (1:1.5, *v*/*v*) and held for 6 min at 25 °C. The mixture was combined with a 10% AlCl_3_ solution and allowed to react for 5 min at 25 °C. The absorbance at 415 nm was compared with rutin hydrate (Sigma Chemical Co.). Dried powder of CLW was extracted with n-butanol saturated with water and filtered to measure the content of crude saponins [16]. Distilled water was added into the filtrates (1:1, *v:v*) and the mixture was vigorously shaken in a separatory funnel. The butanol fraction was separated and concentrated in the vacuum evaporator. The concentrates were mixed with ether and held in a 46 °C water bath for 30 min and then filtered. The precipitates were dried in the desiccator and its weight was measured to be the amount of crude saponins. 

### 2.2. Animals and Ethics 

Male Sprague–Dawley rats (eight weeks of age, 218 ± 23 g) were individually maintained in stainless steel cages in an environmentally-controlled facility (23 °C; 12-h light/dark cycle). All procedures were compliant with the Hoseo University Animal Care and Use Review Committee guidelines (2014-07). After one-week acclimation in the animal facility the rats had a 90% pancreatectomy according to the method of Hosokawa [17] under ketamine and xylazine anesthesia (100 and 10 mg/kg bw).

### 2.3. Experimental Design 

The dosage of CLW was assigned according to our preliminary cell-based study. CLW (20 and 60 μg/mL) enhanced insulin stimulated glucose uptake from 125% to 178% based on the vehicle treatment in 3T3-L1 adipocytes. This was extrapolated to require 300–900 mg CLW/kg bw in an animal study. In consideration of the rat’s food intake and the effective dosage in cell culture, 0.3 and 1% CLW was mixed in a high-fat diet. Px rats were fed their respective diets (43 energy percent) containing 0.3 or 1% a lyophilized powder of *C. lanceolata* (CLW). The 64 Px rats were randomly divided into four groups as follows: (1) 0.3% CLW plus 0.7% cellulose; (2) 1% CLW; (3) 1% cellulose (untreated control); or (4) rosiglitazone (20 mg/kg bw/day) plus 1% cellulose (positive control). The rosiglitazone amount in the positive control diet was calculated according to their food intake on a weekly basis. All rats were freely provided water and assigned diets containing either the assigned CLW or cellulose for eight weeks. The modified semi-purified AIN-93 high-fat-diets [18] were composed of carbohydrate (40%), protein (20%), and fat (45%) from starch and sugar, casein, and lard (CJ Co., Seoul, Korea).

Feed and water consumption and body weights were recorded weekly. After overnight fasting, an oral glucose tolerance test (OGTT) was conducted during the seventh week by giving 2 g glucose/kg body weight [19]. Blood was collected from the animal at 10 min intervals for 90 min and then again at 120 min. Blood glucose concentrations were determined using a Glucose Analyzer II (Beckman-Coulter, Palo Alto, CA, USA). Serum insulin was analyzed with a radioimmunoassay kit at 0, 20, 40, 90 min (Linco Research, Billerica, MA, USA). Serum total and high density lipoprotein (HDL) cholesterol and triglyceride levels were measured using the colorimetry kits (Asan Pharm., Seoul, Korea). Urinary glucose levels were determined using urine glucose test strips (Diastix, Bayer AG, Leverkusen, Germany) every Thursday morning without fasting.

### 2.4. Energy Expenditure, Indirect Calorimetry 

During the seventh week of the experiment, energy expenditure was determined by indirect calorimetry during the dark phase following 6 h of fasting. The rats were placed in a computer-controlled metabolic chamber with the respiratory chamber (800 mL/min airflow) with a computer-monitored O_2_ and CO_2_ system (Biopac Systems Inc., Goleta, CA, USA). The O_2_ consumption (VO_2_) and CO_2_ production (VCO_2_) of the rat were measured every minute for 30 min. The average oxygen consumption (VO_2_) and average carbon dioxide production (VCO_2_) were integrated over periods of 30 min. The respiratory quotient (RQ) was calculated as VCO_2_/VO_2_ and VO_2_ and VCO_2_ values were adjusted for metabolic body size (kg^0.75^) and [20]. Resting energy expenditure (REE) was calculated based on the VO_2_ and RQ [20,21]. Oxidations of fat and carbohydrate calculated from their relative oxidative proportions using RQ [22].

### 2.5. Euglycemic Hyperinsulinemic Clamp

During week 7, hepatic and whole body insulin resistance were estimated using a euglycemic hyperinsulinemic clamp in eight fasted conscious rats at the 5–7th day of the post-catheterization into the jugular vein and carotid artery following previously described procedures [23,24]. Hepatic basal glucose output was determined by infusing 3-^3^H glucose (NEN Life Sciences, Boston, MA, USA) continuously for 2 h at a rate of 0.05 μCi/min and collecting blood at 100 and 120 min. Then, human insulin was continuously infused at a rate of 20 pmol·kg^−1^·min^−1^ which increased the plasma insulin concentration to approximately 1100 pM at 210–240 min. Serum glucose concentrations were constantly held at approximately 6 mM by injecting varied amounts of glucose solution into the jugular vein. Whole-body uptake and basal turnover of glucose were estimated from the ratios of ^3^H glucose infusion rate to specific activity of plasma glucose (dpm/µmol) during the last 30 min of each experiment after measuring wet and dry plasma and tissue (3-^3^H) glucose concentrations [23,24]. Hepatic glucose production during the hyperinsulinemic clamp was determined by subtracting the glucose infusion rate from whole-body glucose uptake [23,24]. 

### 2.6. Hyperglycemic Clamp 

At week 7, catheters were implanted into the right carotid arteries and left jugular veins of all rats under ketamine/xylazine anesthesia. After an overnight fast, hyperglycemic clamps were performed in 10 free-moving rats/group on the 5–6th day post-implantation to estimate the insulin secretory capacity, as described previously [17,25,26]. During the clamp, glucose infusions maintained serum glucose at a concentration of 5.5 mM above baseline. Concentrations of serum insulin were determined at pre-assigned times. Following the clamp, rats allowed free access to feed and water for two days, after which feed was withheld for 16 h. Then the rats were anesthetized with ketamine and xylazine, and blood was collected for serum lipid concentration. The rats were given injections of regular human insulin (5 U/kg body weight; Humulin; Eli Lilly, Indianapolis, IN, USA) into the inferior vena cava. Ten minutes later the rats were decapitated, tissues collected and frozen in liquid nitrogen, and then stored at −70 °C until needed. Triglyceride was extracted with chloroform–methanol (2:1, *v/v*) from the liver and resuspended in chloroform [20]. After removing the chloroform, the residues were suspended with PBS with 0.1% triton X-100 and the suspension was sonicated for 5 min. The hepatic triacylglycerol contents of the suspensions were examined using a Trinder kit (Asan Pharm., Seoul, Korea).

### 2.7. RNA Isolation and Reverse Transcription Polymerase Chain Reaction

Epididymal fat pads from four rats per group were collected post-treatment. The monophasic solutions phenol and guanidine isothiocyanate (Trizol reagent, Gibco-BRL, Rockville, MD, USA) were used to isolate total RNA from the adipose tissues followed by extraction and precipitation with isopropyl alcohol [19]. Then equal amounts of total ribonucleic acid (RNA) were used to synthesize cDNA using superscript III reverse transcriptase. Then polymerase chain reaction (PCR) using high fidelity Taq DNA polymerase was performed. Equal amounts of cDNA were mixed with Sybergreen mix and analyzed using a real-time PCR machine (BioRad, Richmond, CA, USA). The expression levels of genes of interest were indexed to the house-keeping gene, β-actin. Primers were used for detecting rat carnitine palmitoyltransferase (CPT)-1, sterol regulatory element-binding protein (SREBP)-1c, fatty acid synthase (FAS), peroxisome proliferator-activated receptor (PPAR)-γ, Sirtuin (SIRT)-1 and β-actin genes as previously described [19].

### 2.8. Immunoblot Analysis

Liver tissues from six 10 m insulin-stimulated rats were lysed in radioimmunoprecipitation assay (RIPA) lysis buffer containing protease inhibitors. After measuring lysate protein contents with a Bio-Rad protein assay kit (Hercules, CA, USA), lysates with equivalent protein contents (30–50 μg) were resolved by sodium dodecyl sulfate-polyacrylamide gel electrophoresis followed by immunoblotting with antibodies to phosphorylated Akt^ser478^, Akt, phosphorylated glycogen synthase kinase (GSK)-3β, GSK-3β, phosphorylated AMP Kinase (AMPK), AMPK, phosphoenolpyruvate carboxykinase (PEPCK) and β-actin (Cell Signaling Technology, Beverly, MA, USA), [23,24]. The protein expression intensity was determined using Imagequant TL (Amersham Biosciences, Piscataway, NJ, USA). These experiments were replicated three times per group.

### 2.9. Immunohistochemistry

Five rats per group received bromodeoxyuridine injections (BrdU) (100 µg/kg body weight) at the sixth week of treatment. The rats were given anesthesia intraperitoneally 6 h post-injection using ketamine and xylazine, and the pancreases were excised, perfused with saline and a 4% paraformaldehyde solution (pH 7.2) sequentially, and post-fixed at room temperature with the same fixative overnight [27].

Two serial 5-μm paraffin-embedded pancreatic tissue sections were selected from the seventh or eighth sections to avoid counting the same islet twice when measuring β-cell area, BrdU incorporation, and apoptosis by a previously described immunohistochemistry procedure [27]. Endocrine β-cells were identified using antibodies to guinea pig insulin and rabbit glucagon in the sections. Pancreatic β-cell area was measured in two insulin-stained sections from each rat by examining all non-overlapping sections at 10× magnification under a Zeiss Axiovert microscope (Carl Zeiss Microimaging, Thornwood, NY, USA). Pancreatic β-cell mass, individual β-cell size, β-cell proliferation by BrdU incorporation, and apoptotic β-cells were measured as we previously described [27]. Total β-cell area (%) was calculated by the insulin positive area divided by total pancreas area. The individual β-cell size was calculated by the insulin-positive area divided by the number of nuclei counted in the corresponding insulin-positive structures in randomly selected sections. Pancreatic β-cell mass was determined with multiplying the insulin-positive area by the pancreas weight [23,27]. Beta-cell proliferation was calculated as the total BrdU+ nuclei in β-cell nuclei per pancreas section [27]. Apoptosis of β-cells was determined by the total number of apoptotic bodies in β-cell nuclei per pancreas section [27].

### 2.10. Statistical Analyses

All data are expressed as means ± standard deviation. SAS version 9.1 (SAS Institute, Cary, NC, USA) was used for statistical analyses. Significance differences among all groups, animal study and cell-based studies, were assessed by one-way analysis of variance (ANOVA). Differences in the main effects among groups were analyzed using post-hoc Tukey tests. A *p*-value < 0.05 was accepted as significant.

## 3. Results

### 3.1. Total Polyphenols and Flavonoids in CLW

The CLW contained 3956 μg/g of crude saponins, 755 ± 17 μg/g of total polyphenol, 68.3 ± 2.3 μg/g of total flavonoids.

### 3.2. Body Composition and Energy Metabolism

Weight gains and epididymal fat mass were higher in ascending order for the untreated control, CLW-L, CLW-H, and positive control at the end of the experiment (Table 1).

Daily energy intakes were higher in the untreated control group than the CLW-H group, and it was similar to the CLW-H and positive control groups (Table 1). The daily energy expenditure and fat oxidation did not differ significantly among the groups (Table 1). The carbohydrate oxidation was lower in the untreated controls than in the CLW-H and positive control groups (Table 1). Locomotive activity was higher in ascending order for the untreated control group, CLW-L, CLW-H and positive control group (Table 1). The rats in the control were supposed to have higher bodyweights, but the bodyweights of the untreated control group were lower than those of the positive control. The results suggest that the untreated control rats excreted more glucose into the urine than did the positive control and CLW-H rats. Urinary glucose excretion of the untreated control was much greater than the CLW-H and was similar to the positive controls (Table 1).

Lipid profiles in the circulation were disturbed by diabetes: the serum total and low density lipoprotein (LDL) cholesterol and triglyceride levels increased and the serum HDL cholesterol levels decreased in the untreated control group in comparison to the CLW-H (Table 1). CLW prevented dyslipidemia caused by the diabetic condition in a dose-dependent manner and the lipid profiles of CLW-H were similar to the positive control in Px rats (Table 1).

**Figure 1:**
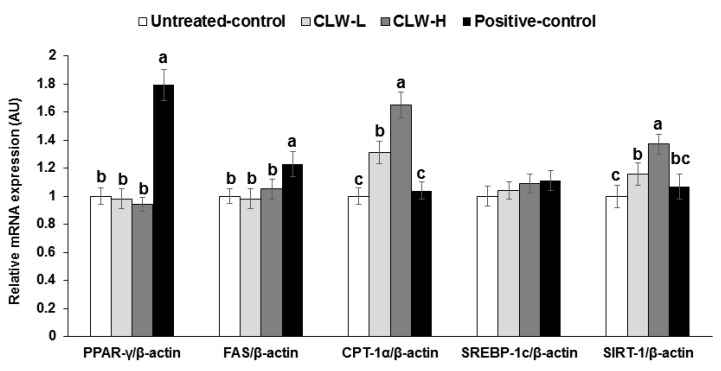
mRNA expression of genes related to fat metabolism in the adipose tissues. Px rats fed a high-fat diet supplemented with 0.3% lyophilized water extract of *C. lanceolata* + 0.7% cellulose (CLW-L), 1% lyophilized water extract of *C. lanceolata* (CLW-H), 1% cellulose (untreated control), or rosiglitazone (20 mg/kg body weight/day; positive control) + 1% cellulose. After 8-week treatment, adipose tissues were collected and cDNA was made from adipose tissues. The mRNA expression of PPAR-γ, CPT-1, SREBP-1c, FAS, and SIRT-1 was measured in the adipose tissues by real-time PCR. Bars and error bars represent means±standard deviation (*n* = 8). ^a,b,c^ Different superscripts on the bars of each variable indicate significant differences in each variable by Tukey test.

Interestingly, in epididymal fat the mRNA expression of PPAR-γ, FAS and SREBP-1c, which are regulatory factors of lipid synthesis, were not different between the untreated control and CLW groups, but they were significantly higher in the positive control group compared to the untreated control group (Figure 1). However, CPT-1 mRNA expression, involved in fatty acid delivery from the cytosol to the mitochondria to be oxidized, was dose-dependently elevated by CLW, in comparison to the untreated and positive control groups (Figure 1). The SIRT-1 mRNA expression involved in the mitochondrial biogenesis showed a similar pattern of CPT-1 mRNA expression (Figure 1).

### 3.3. Glucose Tolerance 

**Figure 2:**
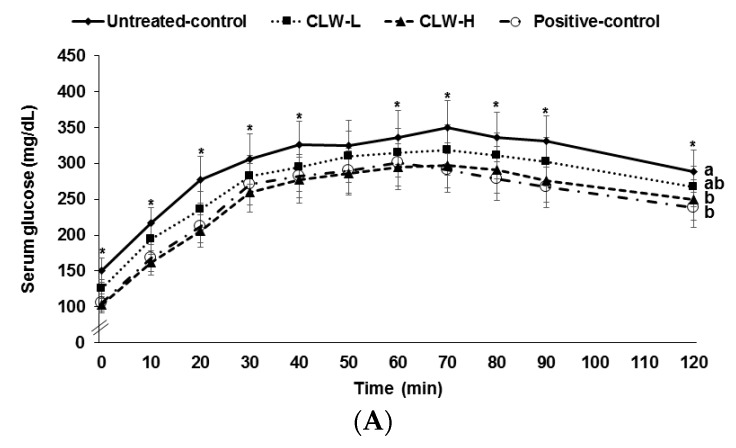

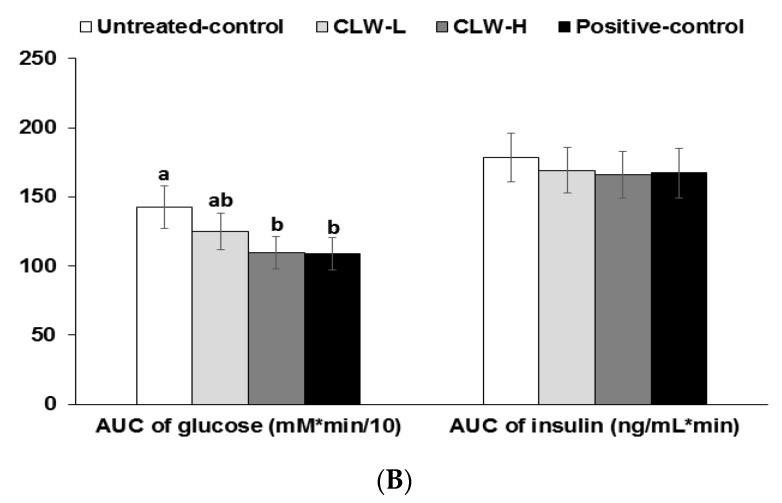
Results of oral glucose tolerance test. Px rats fed a high fat diet supplemented with 0.3% lyophilized water extract of *C. lanceolate* + 0.7% cellulose (CLW-L), 1% lyophilized water extract of *C. lanceolata* (CLW-H), 1% cellulose (untreated control), or rosiglitazone (20 mg/kg body weight/day; positive control) + 1% cellulose. At the seventh week, serum glucose levels were measured every 10 min after oral loading with 2 g glucose per kg body weight (**A**). Serum insulin levels were measured at 0, 20, 40, 80, and 120 min. Area under the curve (AUC) of serum glucose and insulin (**B**) levels were calculated. Dots or bars and error bars represented means±standard deviation (*n* = 16). * Significantly different among the groups in one-way analysis of variance at *p* < 0.05. ^a,b^ Means of the bars without a common letter significantly differ at *p* < 0.05 by Tukey test.

Serum glucose concentrations rose continuously for 60–70 min following the oral glucose challenge and then they slowly decreased (Figure 2A). The concentrations in the CLW-H and positive control rats were lower than in the untreated control rats. Peak serum glucose concentrations were lowest in CLW-H and positive controls (Figure 2A). Areas under the curves (AUC) of serum glucose concentrations were higher in untreated controls than in CLW (Figure 2B). The AUC of serum glucose in the CLW-H group was similar to the positive controls (Figure 2B). The AUC of serum insulin concentrations during OGTT was the same among the groups (Figure 2B).

### 3.4. Insulin Secretion Capacity

**Figure 3:**
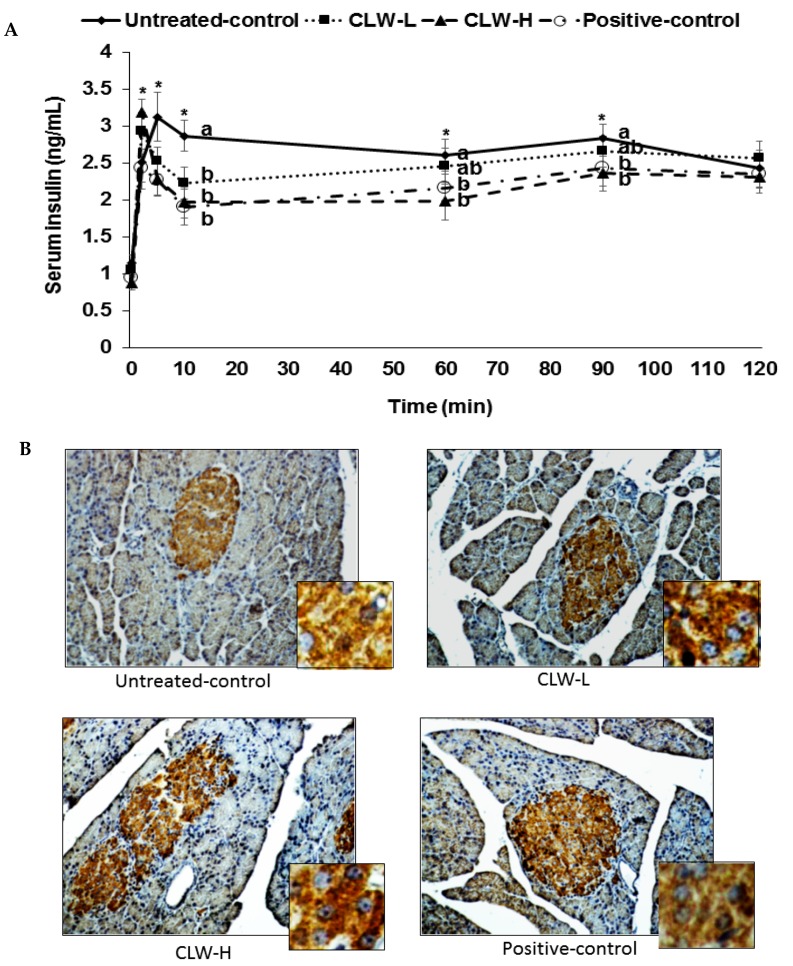
Glucose-stimulated insulin secretion during hyperglycemic clamp. Px rats fed a high-fat diet supplemented with 0.3% lyophilized water extract of *C. lanceolata* + 0.7% cellulose (CLW-L), 1% lyophilized water extract of *C. lanceolata* (CLW-H), 1% cellulose (untreated control), or rosiglitazone (20 mg/kg body weight/day) + 1% cellulose (positive control). At the 8th week serum insulin levels were determined at 0, 2, 5, 10, 60, 90, and 120 min to make serum glucose levels above 5.5 mM from the baseline by infusing glucose solution into the jugular vein (**A**); The insulin-positive-staining (brown) and nucleus (blue) were measured by the pancreas using immunohistochemistry (**B**). The big and small pictures in each group were ×40 and ×400 magnification, respectively. Dots and error bars represent means ± standard deviation (*n* = 8). * Significantly different among the groups in one-way analysis of variance at *p* < 0.05. ^a,b^ Means of the bars without a common letter significantly differ at *p* < 0.05 by Tukey test.

Px rats at zero weeks showed similar fasting serum glucose concentrations, approximately 155 mg/dL, in all groups. Prior to the hyperglycemic clamp assay, the fasting serum glucose and insulin concentrations decreased in CLW in a dose-dependent manner in comparison to the untreated control, and were similar to the positive controls (Table 2). The serum glucose concentrations were sustained at 100 mg/dL above fasting serum glucose levels at 60–120 min by glucose infusions into the jugular vein, and serum insulin levels were determined to assess the insulin secretory capacity. CLW dose-dependently lowered serum glucose levels at 60–120 min compared to the untreated control during the hyperglycemic clamp and the levels were similar in the CLW-H and positive controls (Table 2). The serum insulin concentrations increased at 2–5 min after the infusion of glucose into the jugular vein and then they decreased at 10 min (first phase, Figure 3). Then the serum insulin levels were elevated again and they plateaued from 60 min (second phase, Figure 3). The serum insulin concentrations were lower in the untreated controls than in the CLW-H at 2 min, but they were higher at 5 min. At 10 min, the serum insulin concentrations were much higher in the untreated controls than in the CLW-H (Figure 3). The CLW-H showed a similar pattern of serum insulin levels during the hyperglycemic clamp. This indicated that the untreated control group had impaired insulin secretion patterns compared to the CLW-H and positive control. The AUC of serum insulin concentrations in the first and second phases were highest in the untreated control group as compared to the other groups (Table 2). The glucose infusion rates were much lower in the untreated control than the positive control group, and CLW induced a dose-dependent increase (Table 2). Consistent with the glucose infusion rate, insulin sensitivity at 60–90 min was lower in the untreated control group than the CLW-H group (Table 2). CLW-H had a similar insulin sensitivity in the hyperglycemic state to the positive control.

### 3.5. β-Cell Mass

The area of pancreatic β-cells is estimated by multiplying β-cells numbers by their individual size. The pancreatic β-cell area did not differ between the untreated control and positive control rats, although the size of individual β-cells was larger in the untreated control group than the positive control group (Table 3, Figure 3B). However, CLW dose-dependently elevated the pancreatic β-cell area by decreasing the size and increasing the number of β-cells (Table 3). Pancreatic β-cell mass was estimated by multiplying pancreas weight by β-cell area, revealing that CLW dose-dependently increased β-cell mass compared to the untreated control (Table 3). The β-masses were not significantly different between the CLW-H and positive control groups.

β-Cell apoptosis and proliferation are the major determinants of β-cell numbers. β-Cell proliferation did not differ among the untreated control, CLW and positive control groups (Table 3). Untreated control rats exhibited greater apoptosis of β-cells than CLW, and β-cell apoptosis was similarly suppressed by CLW-H and positive control rats (Table 3), suggesting that CLW can increase β-cell mass by decreasing apoptosis.

### 3.6. Insulin Sensitivity

**Figure 4:**
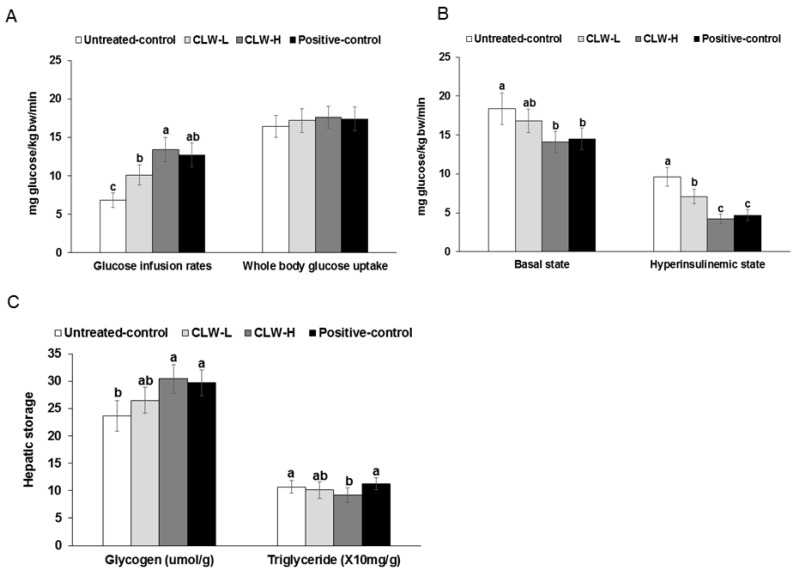
Metabolic parameters during euglycemic hyperinsulinemic clamp. Px rats fed a high-fat diet supplemented with 0.3% lyophilized water extract of *C. lanceolata* + 0.7% cellulose (CLW-L), 1% lyophilized water extract of *C. lanceolata* (CLW-H), 1% cellulose (untreated control), or rosiglitazone (20 mg/kg body weight/day) + 1% cellulose (positive control) for 8 weeks. At the end of the experiment, a euglycemic-hyperinsulinemic clamp was performed in conscious, free-moving, and overnight-fasted rats by infusing insulin into the jugular vein to make 1100 pM serum insulin levels and simultaneously infusing glucose to euglycemia. From the clamp, whole body glucose infusion rates (GIR) and glucose uptake (**A**) and hepatic glucose output at basal and clamped states (**B**) were determined. Glycogen and triglyceride contents in the liver were measured (**C**). Bars and error bars represented means ± standard deviation (*n* = 8). ^a,b,c^ Means of the bars without a common letter significantly differ at *p* < 0.05 by Tukey test.

Glucose infusion rates during the hyperinsulinemic-euglycemic clamp were higher in the CLW-H than the untreated control group, and were similar to the positive control (Figure 4A). The whole-body glucose uptakes were not significantly different among the groups (Figure 4A). The basal hepatic glucose outputs were lower in CLW-H and positive control groups than the untreated control (Figure 4B). Furthermore, hepatic glucose output during the hyperinsulinemic clamp was lower in the descending order of the untreated control, CLW-L, positive control and CLW-H (Figure 4B).

Glycogen storage was elevated in the CLW-H and positive control groups compared to untreated controls (Figure 4C). However, hepatic triglyceride accumulation did not differ significantly except that is was significantly lower in the CLW-H group, compared to the untreated control (Figure 4C).

**Figure 5:**
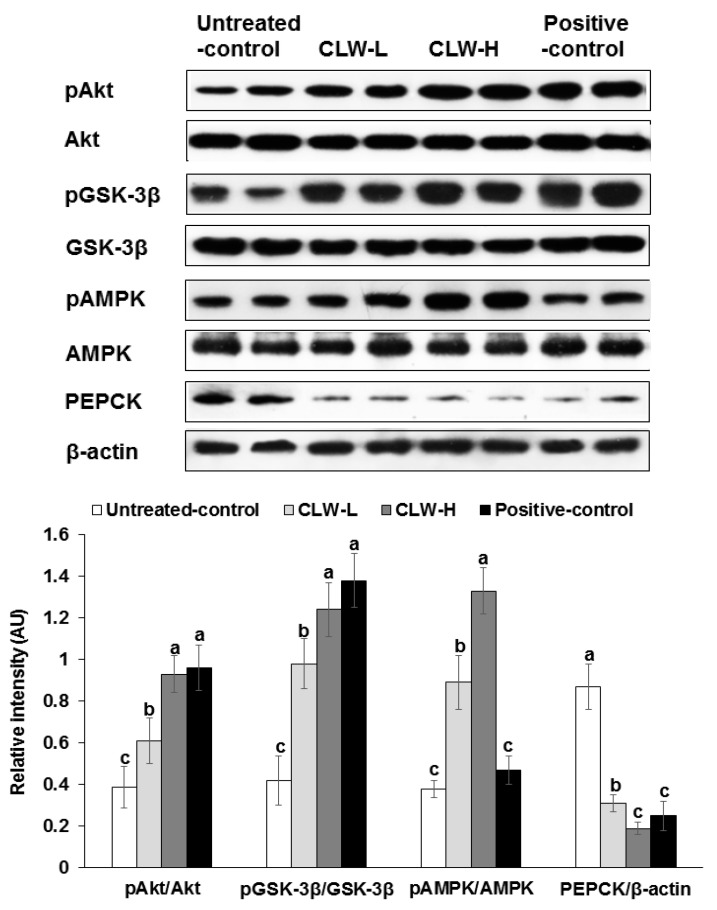
Hepatic insulin signalling. Px rats fed a high-fat diet supplemented with 0.3% lyophilized water extract of *C. lanceolata* + 0.7% cellulose (CLW-L), 1% lyophilized water extract of *C. lanceolata* (CLW-H), 1% cellulose (untreated control), or rosiglitazone (20 mg/kg body weight/day) + 1% cellulose (positive control) for 8 weeks. At the end of experiment, the liver was collected and lysed with radioimmunopreciptation assay buffer. The phosphorylation and expression of proteins related to insulin signaling in the liver lysates were measured by western blot analysis. Bars and error bars represented means ± standard deviation (*n* = 5). ^a,b,c^ Means of the bars without a common letter significantly differ at *p* < 0.05 by Tukey test.

Akt and GSK phosphorylation, which are important for regulating hepatic insulin signaling, were slightly attenuated in the untreated control group compared to the CLW-H and positive control (Figure 5). In addition, PEPCK expression was also higher in the untreated control compared to the positive controls, but CLW-H lowered its expression (Figure 5). The phosphorylation of AMPK was dose-dependently increased by CLW in comparison to the untreated control (Figure 5), but not by the positive control.

## 4. Discussion

We hypothesized that CLW would have a hypoglycemic effect by reducing insulin resistance and tightly regulating insulin secretion in an Asian type 2 diabetic animal model. We examined the efficacy of CLW in treating type 2 diabetic symptoms, and the mechanism by which it improves insulin sensitivity and secretory capacity were explored in male Px rats, a non-obese model of type 2 diabetes. Px rats did not gain as much body weight as the positive control group due to increased urinary glucose excretion by decreased glucose utilization. CLW improved weight gain in a dose-dependent manner, although CLW elevated glucose oxidation and prevented urinary glucose loss as much as the positive control. CLW enhanced glucose tolerance by reducing insulin resistance. The improved insulin sensitivity was associated with hepatic insulin signaling (pAkt➔pGSK-3β), which reduced PECPK expression. Insulin secretion in the second phase was rather lower in CLW than the untreated control. However, β-cell mass was increased in CLW-H compared to the untreated control. Thus, CLW improved the diabetic symptoms by suppressing hepatic insulin resistance by potentiating hepatic insulin signaling as much as rosiglitazone in non-obese type 2 diabetic rats with impaired insulin secretion. This is the first study to show that CLW improves hepatic insulin sensitivity in non-obese type 2 diabetic rats and suggests that it can be used as an intervention for Asian type 2 diabetes.

CLW includes triterpenoid saponins such as lancemaside A, lancemaside B, lancemaside E, and foetidissimoside A [28,29] and these components have not been studied for anti-diabetic effects. Other plant saponins such as ginsenosides in ginseng have been reported to have anti-diabetic effects by potentiating glucose-stimulated insulin secretion and reducing insulin resistance [30]. However, since saponins in CLW and ginseng have different chemical structures and functions, it is difficult to know if terpenoid saponins in CLW are an effective component. However, lancemaside A has been reported to have anti-inflammatory properties by modulating c-Jun N-terminal kinases (JNK) and nuclear factor kappa beta signaling pathways in lipopolysaccharide-induced inflammation [28,31]. In addition, lancemaside A was revealed to ameliorate scopolamine-induced memory deficit in mice. Since inflammation and oxidative stress are associated with the etiology of type 2 diabetes even in non-obese type 2 diabetes and insulin signaling is also interconnected to the JNK signaling pathway [32], Therefore, various lancemasides in CLW may be effective components for alleviating type 2 diabetes.

Following a 90% pancreatectomy, the pancreas partially regenerates back to about 50% of the original mass, with about 50–60% of its original insulin secretory capacity. Px rats gradually develop insulin resistance which accelerates when they are fed high-fat diets [4,33]. Thus high-fat-fed Px rats exhibit the characteristics of type 2 diabetes, with both insulin resistance and impaired insulin secretion (random glucose levels > 180 mg/dL) [17,34]. Effective herbal treatments should potentiate both insulin sensitivity and β-cell function and mass [4,33]. Our results indicated that CLW prevented rampant insulin secretion which exhausts β-cell function and exacerbates insulin resistance. No previous studies have been conducted to examine whether CLW improves hyperglycemia by improving insulin sensitivity and secretion. Previous studies have reported that CLW prevents diet-induced obesity [10,35]. CLW lowers body-fat accumulation by protecting against adipogenesis. However, in the present study both body weight and fat were higher in the CLW supplemented diabetic rats fed a high-fat diet (untreated control group). However, the genes involved in fat synthesis, PPAR-γ, FAS and SREBP-1c were not different in the adipose tissues between the untreated control and CLW groups. CPT-1 mRNA expression was also elevated in CLW. These results suggested that Px diabetic rats had reduced body weight and body fat by suppressing glucose utilization and CLW protected against the impairment of glucose utilization and the increase of substrate supply in the epididymal fat pads in the present study. Although we did not include a normal control group in the present study, previous studies [23,36] have reported that Sham rats in the normal control group increased body weight and body fat more than Px rats, which was associated with the severity of diabetic status. Therefore, the dose-dependent increase of fat mass was associated with the improvement of diabetic symptoms in rats fed CLW. CLW improved diabetic symptoms by improving hepatic insulin signaling and glucose utilization in the adipose tissues.

No study has previously examined the anti-diabetic effects of CLW. The present study demonstrated that CLW dose-dependently enhanced insulin sensitivity, especially in the liver, by potentiating insulin signaling. Although the effect of CLW on hepatic insulin resistance has not been previously examined, some studies have demonstrated that CLW reduced hepatic steatosis from chronic alcohol consumption [37,38] and it decreases hepatic lipid accumulation by increasing hepatic and lipoprotein lipase activity [6]. CLW is reported to accelerate lipid metabolism and to reduce lipid accumulation in the tissues including the liver and adipocytes. This may be related to potentiating insulin signaling and AMPK activation. The present study also demonstrated that insulin signaling (pAkt➔pGSK ➔ PEPCK) and the phosphorylation of AMPK was enhanced by CLW in a dose-dependent manner in the liver and, as a result, CLW-H decreased triglyceride contents. The phosphorylation of AMPK has been shown to increase SIRT-1 mRNA expression [39]. Triglyceride storage was elevated in the positive controls compared to the CLW-H, but not as compared to the untreated control group. In addition, the glycogen storage was significantly lower in the untreated control group than the CLW-H and positive control groups. Thus, the improved hepatic insulin sensitivity was associated with potentiated hepatic insulin signaling, which resulted in tight regulation of hepatic glucose output due to decreased PEPCK. 

In addition to hepatic insulin sensitivity, CLW improved whole-body insulin sensitivity compared to the untreated control, as determined by the euglycemic hyperinsulinemic clamp, despite the increasing fat mass in the present study. The decrement of fat mass in the untreated control group itself did not improve insulin sensitivity. The improved insulin sensitivity by CLW might be associated with increased SIRT-1 expression in the adipose tissues in comparison to the untreated control. SIRT-1 functions as an intracellular metabolic regulator by affecting peroxisome proliferator-activated receptor gamma coactivator 1-α/estrogen-related receptor-α complex and is involved in insulin sensitivity [40,41]. Furthermore, SIRT-1 is reported to promote fat mobilization, increase mitochondrial size and number, and regulate insulin secretion [42]. CLW may stimulate SIRT-1 activity by increasing SIRT-1 expression to improve insulin sensitivity in the adipose tissues.

There were some limitations in the present study. First, the animal model was Px rats with non-obese type 2 diabetes with normoinsulinemia or hypoinsulinemia, which has similar characteristics to Asian type 2 diabetes. The animal model exacerbated insulin resistance with a high-fat diet. The lean type 2 diabetic animal model is different from the obese type 2 diabetic animal model. The lean type 2 diabetic animal model lowered body weight, which is different from obesity-related type 2 diabetes, which is the most common in Western countries. CLW improved insulin signaling and it prevented the loss of bodyweight in non-obese type 2 diabetic rats, although it may protect against weight gain in obese type 2 diabetes, thereby by leading to normoinsulinemia. Second, sham-operated rats were not included as the normal control to show the improvement of diabetic severity by CLW in Px rats. However, previous studies [20,30] have shown that weight loss with decreasing fat mass represents severe diabetic status and the protection against weight loss is one of indicators of improved diabetic status in Px rats, which is similar to Asian diabetic patients. Finally, this study did not evaluate the individual components in the extract which would be useful information to obtain in the future for optimizing the extracts for greater safety and efficacy. 

## 5. Conclusions

Px diabetic rats exhibited lower bodyweight and body fat due to increased urinary glucose loss and decreased glucose utilization. CLW increased bodyweight gain, which was associated with markedly decreased urinary glucose excretion and enhanced glucose utilization, and with increased insulin sensitivity and better regulation of insulin secretion. This was related to the efficacy of CLW and the positive control group to reduce diabetic severity by improving glucose utilization in the epididymal fat and liver. CLW improved the diabetic diagnostic criteria by reducing hepatic insulin resistance by potentiating hepatic insulin signaling (pAkt➔pGSK-1β) and lowering hepatic triglyceride contents by increasing the phosphorylation of AMPK in non-obese type 2 diabetic rats. CLW exhibited a tight regulation of the insulin secretory capacity to protect against the exhaustion of β-cell function. Thus, CLW can be a useful herbal treatment for type 2 diabetic patients who are not obese, but have insufficient insulin response to normalize blood glucose when insulin resistant. 

## Figures and Tables

**Table 1:** Energy metabolism and lipid profiles at the end of the experimental periods.

	Untreated Control (*n* = 16)	CLW-L (*n* = 16)	CLW-H (*n* = 16)	Positive Control (*n* = 16)
Body weight (g)	326 ± 19 ^b^	342 ± 29 ^a,b^	351 ± 37 ^a^	347 ± 28 ^a^
Epididymal fat pads (g)	2.8 ± 0.6 ^b^	3.5 ± 0.7 ^a,b^	4.0 ± 0.7 ^a^	3.8 ± 0.7 ^a^
Caloric intake (kcal/day)	87.7 ± 7.6 ^a^	84.6 ± 7.8 ^a,b^	80.2 ± 8.1 ^b^	79.2 ± 8.4 ^b^
Intake of *C. lanceolata* (mg/day/kg bw)	0 ± 0 ^c^	161 ± 17 ^b^	746 ± 73 ^a^	0 ± 0^c^
Caloric expenditure (kcal/kg bw^0.75^/day)	104 ± 11	106 ± 13	114 ± 15	109 ± 12
Carbohydrate oxidation (mg/kg bw^0.75^/min)	3.3 ± 0.5 ^b^	3.6 ± 0.5 ^b^	4.3 ± 0.6 ^a^	4.3 ± 0.5 ^a^
Fat oxidation (mg/kg bw^0.75^/min)	7.8 ± 0.9	7.6 ± 0.9	7.9 ± 0.9	7.4 ± 0.8
Locomotive activity (cm/min)	60.6 ± 8.3 ^c^	71.5 ± 9.5 ^b^	80.2 ± 9.7 ^a^	77.8 ± 8.9 ^a,b^
Urinary glucose	++++	++	+	+
Total cholesterol (mg/dL)	101.7 ± 10.2 ^a^	99.2 ± 10.5 ^a^	93.3 ± 9.6 ^a,b^	90.8 ± 9.7 ^b^
HDL cholesterol (mg/dL)	22.2 ± 2.7 ^b^	24.1 ± 2.6 ^a,b^	25.9 ± 2.4 ^a^	26.3 ± 2.6 ^a^
LDL cholesterol (mg/dL)	62.8 ± 5.9 ^a^	62.4 ± 6.8 ^a^	56.7 ± 6.1 ^b^	52.3 ± 5.6 ^b^
Triglyceride (mg/dL)	78.4 ± 8.1 ^a^	63.6 ± 6.9 ^b^	53.6 ± 5.7 ^c^	60.9 ± 6.6 ^b^

Values are means ± standard deviation. Px rats fed a high-fat diet supplemented with 0.3% lyophilized water extract of *C. lanceolata* + 0.7% cellulose (CLW-L), 1% lyophilized water extract of *C. lanceolata* (CLW-H), 1% cellulose (untreated control), or rosiglitazone (20 mg/kg body weight/day; positive control) + 1% cellulose for 8 weeks. ^a,b,c^ Values on the same row with different superscripts were significantly different at *p* < 0.05. Values for urinary glucose excretion are expressed as relative values and not numerical values since that is how the values are indicated on the glucose measurement strips, and statistical comparisons cannot be calculated.

**Table 2:** Insulin secretion capacity during the hyperglycemic clamp.

	Untreated Control (*n* = 8)	CLW-L (*n* = 8)	CLW-H (*n* = 8)	Positive Control (*n* = 8)
Overnight-fasted serum glucose (mg/dL)	151 ± 18 ^a^	125 ± 17 ^b^	101 ± 13 ^c^	102 ± 16 ^c^
Overnight-fasted serum insulin (ng/mL)	1.14 ± 0.12 ^a^	1.05 ± 0.09 ^a,b^	0.88 ± 0.10 ^b^	0.94 ± 0.11 ^b^
Serum glucose at 60–120 min (mg/dL)	254 ± 12 ^a^	230 ± 10 ^b^	208 ± 9 ^c^	207 ± 10 ^c^
AUC of serum insulin at first phase (ng/mL min)	22.0 ± 2.9 ^a^	17.9 ± 1.9 ^b^	15.5 ± 1.8 ^c^	14.8 ± 1.6 ^c^
AUC of serum insulin at second phase (ng/mL min)	231 ± 24 ^a^	206 ± 22 ^b^	168 ± 18 ^c^	176 ± 19 ^c^
Glucose infusion rate (mg/kg bw/min)	7.3 ± 1.1 ^b^	8.1 ± 1.2 ^b^	9.5 ± 1.5 ^a^	9.8 ± 1.4 ^a^
Insulin sensitivity (µmol glucose min^−1^ 100 g^−1^ per µmol insulin/L)	9.5 ± 1.6 ^c^	11.8 ± 2.0 ^b^	17.0 ± 2.6 ^a^	16.7 ± 2.3 ^a^

Values are means ± standard deviation. Px rats fed a high-fat diet supplemented with 0.3% lyophilized water extract of *C. lanceolata* + 0.7% cellulose (CLW-L), 1% lyophilized water extract of *C. lanceolata* (CLW-H), 1% cellulose (untreated control), or rosiglitazone (20 mg/kg body weight/day; positive control) + 1% cellulose for 8 weeks. ^a,b^^,c^ Values on the same row with different superscripts were significantly different at *p* < 0.05.

**Table 3:** The modulation of islet morphometry.

	Untreated Control (*n* = 8)	CLW-L (*n* = 8)	CLW-H (*n* = 8)	Positive Control (*n* = 8)
Total β-cell area (%)	5.7 ± 0.6 ^c^	6.8 ± 0.9 ^b^	8.4 ± 1.0 ^a^	7.3 ± 0.9 ^b^
Individual β-cell size (μm^2^)	238 ± 30 ^a^	216 ± 27 ^a,b^	195 ± 27 ^b^	187 ± 26 ^b^
Absolute β-cell mass (mg)	17.1 ± 2.8 ^c^	21.9 ± 3.9 ^b^	27.7 ± 3.7 ^a^	25.2 ± 3.2 ^a^
BrdU^+^ cells (% BrdU^+^ cells of islets)	0.87 ± 0.10	0.91 ± 0.11	0.94 ± 0.11	0.92 ± 0.12
Apoptosis (% apoptotic bodies of islets)	0.82 ± 0.09 ^a^	0.64 ± 0.08 ^b^	0.53 ± 0.07 ^c^	0.59 ± 0.08 ^b,c^

Values are means ± standard deviation. Px rats fed a high-fat diet supplemented with 0.3% lyophilized water extract of *C. lanceolata* + 0.7% cellulose (CLW-L), 1% lyophilized water extract of *C. lanceolata* (CLW-H), 1% cellulose (untreated control), or rosiglitazone (20 mg/kg body weight/day; positive control) + 1% cellulose for 8 weeks. ^a,b,c^ Values on the same row with different superscripts were significantly different at *p* < 0.05.

## Abbreviations

CLW	Codonopsis lanceolata water extract
Px	pancreatectomized
OGTT	oral glucose tolerance test
VO_2_	O_2_ consumption
VCO_2_	CO_2_ production
REE	resting energy expenditure
RQ	respiratory quotient
CPT	carnitine palmitoyltransferase
SREBP	sterol regulatory element-binding protein
FAS	fatty acid synthase
PPAR	peroxisome proliferator-activated receptor
BrdU	bromodeoxyuridine
SIRT	Sirtuin
GSK	glycogen synthase kinase
AUC	areas under the curve
AMPK	AMP Kinase
JNK	c-Jun N-terminal kinases
